# Notes and News

**Published:** 1904-11-12

**Authors:** 


### NOTES AND NEWS.

THE Charity Organisation Society has taken up the question of the provision of consumption sanatoria.

THE calling of the sanitary inspector is not without danger. At Oldbury two butchers have been heavily fined for assaulting the sanitary inspector and the medical officer who accompanied constables to seize meat upon their premises.

WE regret to announce the death of Mr. H. W. Allingham, F.R.C.S., which occurred at Marseilles on the 4th inst. Mr. Allingham, who was on the staff of St. George's Hospital, was one of the most successful surgeons of the day; for, although comparatively young, he was Surgeon to the Household of his Majesty and Surgeon in Ordinary to the Prince of Wales, and held also other distinguished offices in the profession.

THE Duke of Norfolk is a warm advocate for the establishment of indoor miniature rifle ranges, and he is interesting himself in the establishment of these through the agency of the Society of Miniature Rifle Clubs, which has its headquarters at 20 Bucklersbury, Queen Victoria Street, E.C. In the winter months any organised amusements with useful aims are to be welcomed, and it is to be hoped that many localities will take advantage of the Society's assistance to establish these clubs, which are bound to be popular amongst the youth of the metropolis.

AMONGST the Birthday Honours List the following names appear:—Dr. Charles H. Marriott, Dr. Shirley Murphy, Esq., Major Allan Perry, M.D., Principal Civil Medical Officer and Inspector-General of Hospitals in Ceylon, and Professor W. Sinclair are created Knights. Francis Watts, Esq., Analytical and Agricultural Chemist for the Leeward Islands, is created C.M.G. Dr. C. Marriott was many years consulting surgeon at the Leicester Infirmary; Dr. Shirley Murphy is medical officer to the London County Council; Professor Sinclair is Professor of Obstetrics and Gynæcology at the Manchester Infirmary.

WE are glad to learn that the urgent appeal made by the North-Eastern Hospital for Children is being actively supported by the clergy in the district. Those whose work lies amongst the poor, who depend on the hospital to assist their sick children, know what a calamity it will be if some of the wards have to be closed. It is not possible that the necessary support can come from Bethnal Green, and so it is to be hoped that generous friends will be forthcoming from other sources.

THE protest against the number of nursing homes in the neighbourhood of Harley Street still continues; a writer in a local paper remarks.

> "If it be necessary to have in this parish a colony of the infirm, the sick and the moribund, is it not reasonable that a certain quarter should be exclusively allocated for that purpose so that there should be no private residents to annoy? Why should residences in various parts of this parish be transformed into hospitals? We hear of a grocer's establishment having on the same premises a nursing home; shall we soon hear of the butcher and the baker having similar institutions on their premises? Let us hope that, for the good of the community, we shall not."

A SESSIONAL meeting of the Sanitary Institute will be held in the Guildhall, Nottingham, on Saturday, November 26th, at 11 A.M., when a discussion on the "Present-Day Aspect of Conservancy Systems will be opened by Philip Boobbyer, M.D., M.S. M.R.C.S. In the afternoon visits will be made to municipal undertakings illustrative of sanitary practice and administration. A conference on school hygiene is arranged by the Institute to be held in the University of London for February, 1905. Sir Arthur Rücker has consented to act as president.

THE late Mrs. Selina Clark, of Earl's Court, has bequeathed £1,000 each to the Brompton Hospital for Consumption and the London Hospital, and £500 each to the Brompton Cancer Hospital, St. Mary's, the Middlesex, University College, the Royal Free, King's College, Charing Cross, St. George's, and the Westminster Hospital. The London Fever Hospital, the British Home for Incurables, the Royal Hospital for Incurables, and the Kensington Dispensary all benefit to a smaller extent; while Mrs. Clark's residuary estate will be distributed between Chelsea Hospital for Women, the Victoria Hospital for Children, and one or two other charities.

THE County Councils of England and Wales, the Corporation of the City of London, the Councils of County Boroughs and Metropolitan Boroughs and of the Metropolitan Asylums Board met in conference on Thursday to consider the spread of infectious disease by vagrants. Alderman Newton of Newcastle who moved the first motion, is of the opinion that the evil can be dealt with by conferring further powers upon the local sanitary and other authorities. Dr. M. Young, Medical Officer of Health for Stockport, holds that the transmission of smallpox can be prevented by the co-operation of Boards of Guardians and sanitary authorities throughout the country. On the question of Common Lodging Houses and Casual Wards, Alderman Newton suggests that in times of epidemic the sanitary authorities should have increased control over the keepers of these, and over the inmates, and he specifies the extent he recommends.

